# Characterizing burnout among healthcare epidemiologists in the early phases of the COVID-19 pandemic: A study of the SHEA Research Network

**DOI:** 10.1017/ash.2023.124

**Published:** 2023-03-17

**Authors:** Tucker John Guy Smith, Rachel Pryor, Susy S. Hota, Sarah D. Haessler, Valerie M. Deloney, Gonzalo Bearman

**Affiliations:** 1 Virginia Commonwealth University School of Medicine, Richmond, Virginia, United States; 2 Hospital Infection Prevention Program, Virginia Commonwealth University Health System, Richmond, Virginia, United States; 3 North Hospital, Virginia Commonwealth University Health System, Richmond, Virginia, United States; 4 Division of Infectious Diseases, Department of Medicine, University of Toronto, Toronto, Ontario, Canada; 5 Division of Infectious Diseases, Department of Medicine, University of Massachusetts Medical School–Baystate, Springfield, Massachusetts, United States; 6 Society for Healthcare Epidemiology of America (SHEA), Arlington, Virginia, United States; 7 Division of Infectious Diseases, Department of Medicine, Virginia Commonwealth University, Richmond, Virginia, United States

## Abstract

A multisite research team proposed a survey to assess burnout among healthcare epidemiologists. Anonymous surveys were disseminated to eligible staff at SRN facilities. Half of the respondents were experiencing burnout. Staffing shortages were a key stressor. Allowing healthcare epidemiologists to provide guidance without directly enforcing policies may improve burnout.

Healthcare worker burnout is defined by emotional and physical exhaustion, inefficiency, and negative thoughts about work.^1^ Drivers of burnout include work overload, diminished autonomy, and conflicting values with coworkers and administrators. Burnout is a significant challenge to healthcare with dire implications; higher rates of burnout are related to worsening patient safety and quality of care.^2,3^

Burnout studies typically focus on frontline healthcare workers. The literature lacks burnout data among healthcare epidemiologists, experts in patient safety, and hospital-associated infection prevention. Healthcare epidemiologists offer guidance to hospital leaders, but many are not positioned to decide on final policies, nor can they be responsible for policy enforcement. They can experience psychological distress when issuing policies that affect thousands of people^4^ and a sense of nonmembership from the physician community. Burnout in this subspecialty may lead to dual repercussions, affecting healthcare epidemiologists directly and the healthcare personnel and patients influenced by their work^1^. A survey was proposed to address burnout among healthcare epidemiologists.

## Methods

The SHEA Research Network (SRN) is a consortium of 95 healthcare facilities from 19 countries and 35 states that conducts multicenter research in healthcare epidemiology. Researchers proposed an electronic survey–based project to the SRN to assess attitudes of physicians working in healthcare epidemiology. Survey questions were developed by adapting the Physician Worklife Study, MEMO Study, Healthy Workplace Study, and The Mini Z using Alchemer software. A convenience sample was utilized. Principal investigators from SRN facilities were sent email surveys 4 times, and they were invited to forward the survey to their colleagues. SRN facility ID codes were not collected. Responses remained anonymous. A definitive response rate was not calculated.

## Results

In total, 65 surveys were completed, and 88% of respondents worked at teaching hospitals and spent most of their clinical time in inpatient settings (Table 1). Among them, 58% spent at least half of their time on healthcare epidemiology. Also, 51% were experiencing a surge of COVID-19 cases at the time they completed the survey.

**Table 1.** Survey Demographics of Healthcare Epidemiologists

Characteristic	Total (N=65), No. (%)
**Sex**	
Female	35 (54)
Male	29 (45)
Declined to answer	1 (2)
**Race**	
White	55 (85)
Asian	6 (9)
Other	2 (3)
Declined to answer	2
**Years since completing training**	
<1 y	1 (2)
1–5 y	9 (14)
5–10 y	11 (17)
10–20 y	29 (45)
20 or more	15 (23)
Mean y	14.6
**Degree held**	
MD or DO	64
NP (nurse practitioner)	1
**Setting for majority of clinical time**	
Inpatient	56 (86)
Outpatient	8 (12)
Not available	1
**Approximate time spent on healthcare epidemiology**	
0–25%	13 (20)
25–50%	14 (22)
50–75%	15 (23)
75–100%	23 (35)
Mean %	52.2
**Teaching hospital**	
Teaching hospital	57 (88)
Nonteaching hospital	8 (12)
**Hospital experiencing surge of COVID-19 cases at time of response**	
Yes	33 (51)
No	32 (49)

**Fig. 1.**
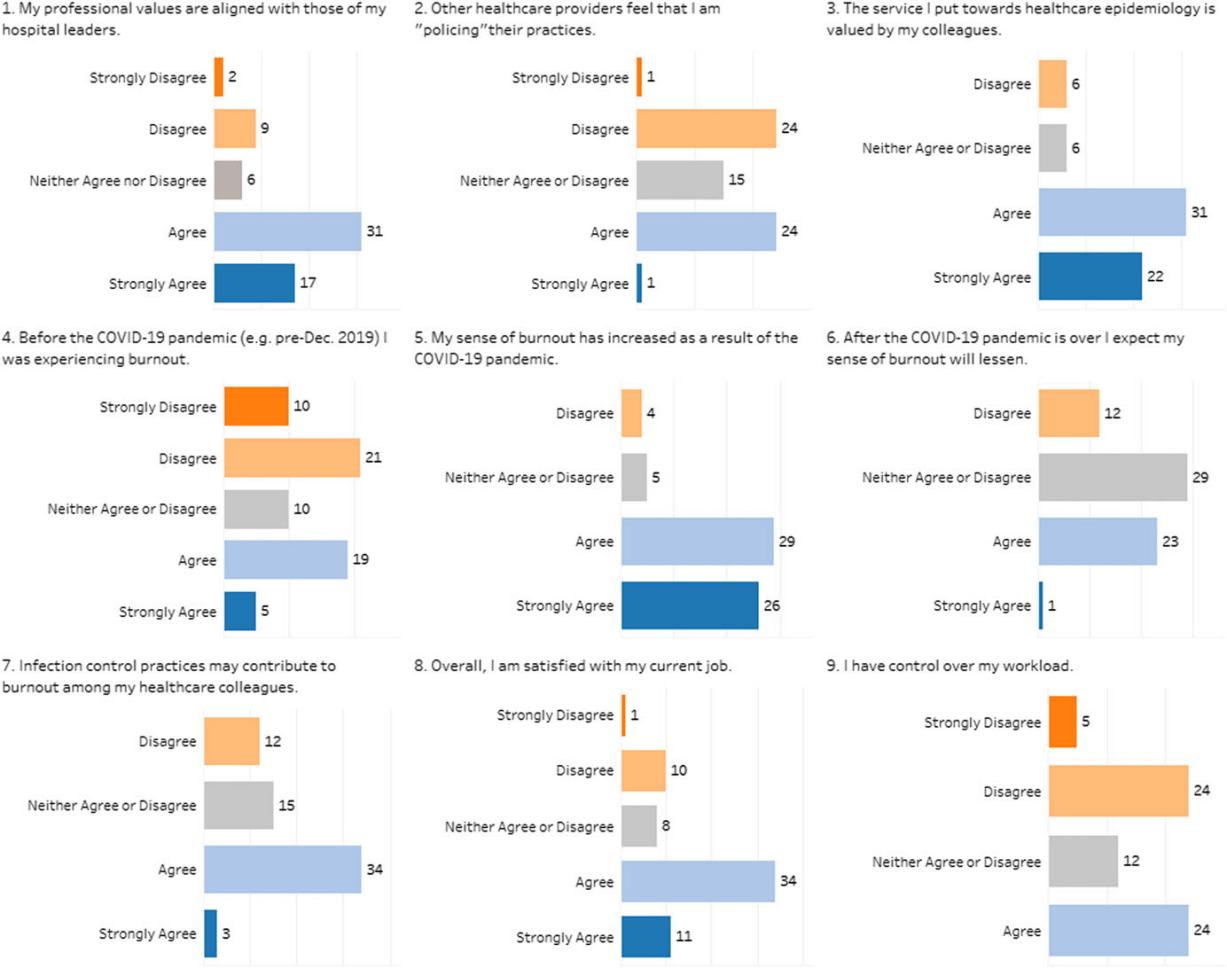
Likert-scale responses to burnout survey.

Most respondents (71%) felt that their professional principles align with those of hospital leaders. Furthermore, 38% believe that, in the eyes of their healthcare personnel (HCP) colleagues, healthcare epidemiologists play a “policing” role in the healthcare setting. Using their own definition, 50.7% of respondents reported that they were experiencing burnout. In addition, 45% reported feeling that they lack control over their workload, and 86% reported their sense of burnout increased as a result of the COVID-19 pandemic. Of those experiencing burnout, 1 in 10 agreed that they were completely burned out and may need to seek help. Figure 1 illustrates responses to survey questions.

In an open-response section, healthcare epidemiologists were asked to identify work stressors and solutions to burnout; 36 comments were collected. Common stressors included staffing shortages, COVID-19 surges, inefficient methods of implementing infection prevention (IP), feelings that healthcare epidemiologists are tasked with holding other HCP accountable for adherence to IP practices, and conflicting guidance from government health agencies throughout the pandemic. Solutions ranged from increasing staffing and resources for IP (eg, improved IP technology), prioritization of work–life balance, and additional “back-up” from hospital administration on implemented policies. Some respondents emphasized the importance of research to demonstrate the need for improvements in IP departments. One responder noted that policy guidance and advocacy from organizations like SHEA have been beneficial. Despite high levels of self-reported burnout, 66.1% of respondents felt valued by their colleagues, and 70% reported job satisfaction.

## Discussion

Healthcare epidemiologists serve as leaders of infection prevention while playing key advisory roles during outbreaks and pandemics. Consequences of burnout in healthcare epidemiologists are concerning; burnout directly affects epidemiologists with downstream effects on healthcare personnel (HCP) and patients. The SRN facilitates the evaluation of perspectives among healthcare epidemiologists. Likert-scale survey questions and open responses facilitate quantitative and qualitative analyses of burnout. Half of the respondents were experiencing burnout, mirroring the overall burnout prevalence among HCP during the COVID-19 pandemic.^5^ Most respondents experienced job satisfaction and had a sense that colleagues value their work. These protective factors may help mitigate the effects of burnout.

More than one-third of respondents believe that they are “policing” other healthcare providers in the eyes of their colleagues, and 58% believe that infection prevention (IP) practices contribute to burnout in their HCP colleagues. Maximizing healthcare epidemiologists’ work within their area of expertise (ie, providing IP guidance as opposed to directly enforcing it) may reduce burnout by decreasing the burden of enforcement, minimizing the feeling that they are directly contributing to HCP burnout, and improving their perception by colleagues. One example of support involves procuring healthcare technology that allows for enhanced data collection and indirect monitoring of IP practices.

Control of workload emerged as a contributor to burnout in nearly half of the respondents. Prior to the pandemic, healthcare epidemiologists faced growing responsibilities, known as responsibility creep.^6,7^ Expansion of hospital systems to multiple locations has increased demands on healthcare epidemiologists, and free-response data indicated that staffing shortages were significant stressors. Overwork emerged as another major stressor that appears to be a consequence of inadequate adjustments in staffing for IP teams prior to the COVID-19 pandemic. During the COVID-19 pandemic, respondents noted a lack of necessary bolstering of IP teams as well, which negatively affected the implementation of IP policies. In addition, 41% said that they were not experiencing burnout prior to the pandemic, yet 86% reported that the COVID-19 pandemic exacerbated feelings of burnout. Stressors related to the pandemic response reflected those indicated in previous studies, including increases in hospital-acquired infections,^8^ continuously changing guidelines, and dissonant guidance from government agencies and professional organizations, leading to a reduced sense of autonomy.^9,10^ As the responsibilities of healthcare epidemiologists grow due to responsibility creep, pandemic response, and the threat of emerging infectious diseases, chronic understaffing of IP teams will continue to drive overwork and burnout among this employee population, 10% of whom are already at critical burnout levels based on survey findings.

Respondents proposed solutions to burnout ranging from internally focused efforts such as improved work–life balance to the externally focused solutions of increased support from administration and research to demonstrate the need for adequately resourced IP teams. Bilateral collaboration between healthcare epidemiologists and public health entities may help improve relationships, build trust, and reduce stress. Improved staffing and greater accountability of safety practices by management allow healthcare epidemiologists to utilize their expertise most effectively toward patient safety, decreasing feelings of overwork and negative perceptions as “hospital police” from their colleagues.

In conclusion, to preserve the healthcare epidemiologist workforce, it is imperative to consider the harmful and protective factors related to burnout. Even as overwork, chronic understaffing and the effects of the pandemic stand out as primary contributors to burnout, healthcare epidemiologists feel valued by colleagues and are satisfied with their jobs. Health systems that empower healthcare epidemiologists to work within their areas of expertise may see decreased rates of burnout and better-functioning IP departments, which would contribute to protecting against emerging infectious diseases and antimicrobial resistance. Failing to meet the needs of IP departments and/or to address burnout leaves healthcare organizations and society vulnerable to large-scale health crises, daily safety risks, and negative effects on healthcare operations and delivery.

This study had several limitations. It had a small sample size and overrepresentation of inpatient healthcare epidemiologists at academic centers. Follow-up studies could include assessing burnout rates and work perceptions among outpatient healthcare epidemiologists and those working at nonacademic centers.
